# Ask not liberal or conservative intravenous fluids in septic shock: ask rather why and when

**DOI:** 10.1186/s13049-022-01054-w

**Published:** 2022-12-08

**Authors:** Jon-Emile S. Kenny

**Affiliations:** Health Sciences North Research Institute, 56 Walford Rd, Sudbury, ON P3E 2H2 Canada

We are familiar with the clinical signs that animate us to administer intravenous (IV) fluids in sepsis and septic shock. Fundamentally, these features—hypotension, oliguria, confusion, skin mottling, prolonged capillary refill, elevated lactate, etc.—are surrogates for hypoperfusion, that is, low blood flow [1, 2]. Hence, the implicit, intended-effect of IV fluid is to raise blood flow and the mechanism of this intended-effect is the Frank-Starling principle [1]. The rub is that we uncommonly measure how blood flow changes in response to IV fluid [3]. As a consequence, we rely on the aforementioned clinical surrogates as imperfect guides [1].

To be more concrete, let us consider blood pressure. In a simplified model of the circulatory system, blood pressure is the product of blood *flow* and vascular resistance. Therefore, for a given resistance, increasing flow from the heart raises pressure and from this relationship we leverage blood pressure to adjacently inform us on blood flow. But what about the not-infrequent scenario where IV fluid is given and blood pressure changes little, if at all? Is this because blood flow did not budge? Or did flow indeed rise, but in the face of falling resistance?[1] Without measuring the intended-effect of IV fluid, the clinician cannot definitively answer these clinical questions. On the other hand, evaluating the heart’s flow response to a fluid challenge resolves this dilemma and directs management. For example, if the heart is flow unresponsive—or ‘refractory’ [4]—to IV fluid, then additional boluses can be safely withheld [5, 6] and other interventions to raise perfusion attempted.

But how common is the heart refractory to IV fluids in sepsis and septic shock? With the available evidence, the answer is that it is surprisingly common. In the landmark ANDROMEDA-SHOCK investigation [7], which is the largest to date wherein the heart’s ability to respond to IV fluid in septic shock was systematically measured, approximately 30% of patients were fluid refractory following an initial bolus (i.e., 20 mL/kg of IV fluid). Then, during an 8-hour resuscitation protocol, the fraction of fluid refractory patients gradually increased to over 90%! The FRESH study [6], a 72-hour resuscitation protocol comparing usual care to fluid provision based on the heart’s flow response, found that nearly 60% of patients were fluid refractory at least once and roughly 1-in-5 were *persistently* fluid refractory across the entire 72-hour investigation. Similar observations were made by Chen and Kollef [8] who reported that over 2/3rds of septic shock patients were fluid refractory on day 1, 88% on day 2 and 100% on day 3. Given these data, it is possible that during septic shock the majority of patients administered IV fluid are, in fact, refractory to this intervention [5, 6, 8].

Where does this leave us within the context of ‘liberal’ versus ‘conservative’ fluid management approaches? While both the CLASSIC [9] and forthcoming CLOVERS [10] evaluations are true milestones in critical care research, they both offer, at best, a ‘two sizes fit all’ view of IV fluid delivery. They do not address whether an individual patient will—or, quite commonly, will not—have the intended-effect of IV fluid resuscitation at any given time and over the arc of illness. Might fluid refractory patients benefit from a restrictive, conservative approach? Will patients who respond appropriately to IV fluid benefit from—or not be harmed by—a liberal approach? We do not know. Of course, there are hints in subgroup analyses. 65% of patients in CLASSIC had received ≥ 30 mL/kg of IV fluid at randomization. This subgroup is likely to have concentrated fluid refractory patients and there was a trend towards lower 90-days mortality for those in this subgroup randomized to the restrictive paradigm. Conversely, in the 35% of patients who had *not* received 30 mL/kg at randomization, there was likely overrepresentation of fluid responsive patients; in this subgroup, those randomized to the liberal arm trended towards lower mortality. Of course, these observations are hypothesis-generating at best. Nevertheless, the aforementioned FRESH [6] study gives credence to the premise of restricting IV fluid in refractory patients. In FRESH, patients with septic shock who were randomized to receive IV fluid therapy based upon the heart’s flow-response received *less* IV fluid and had fewer fluid-related complications as compared to usual care. Ostensibly, this occurred because cryptic fluid refractory states were detected and IV fluid restricted at the appropriate time [4, 6].

We should look forward to the forthcoming results of ANDROMEDA-SHOCK 2 and others who attempt resuscitation based on hemodynamic phenotypes. Much as we tailor antimicrobials to culture and sensitivity, bespoke IV fluid founded upon cardiac preload sensitivity should shape septic resuscitation strategy in the 21st century.

## Data Availability

Not applicable.
